# Exploration of anti-stress mechanisms in high temperature exposed juvenile golden cuttlefish (*Sepia esculenta*) based on transcriptome profiling

**DOI:** 10.3389/fphys.2023.1189375

**Published:** 2023-05-10

**Authors:** Yongjie Wang, Xiaokai Bao, Weijun Wang, Xiaohui Xu, Xiumei Liu, Zan Li, Jianmin Yang, Tingzhu Yuan

**Affiliations:** ^1^ School of Agriculture, Ludong University, Yantai, China; ^2^ College of Life Sciences, Yantai University, Yantai, China; ^3^ Marine Economy Promotion Center of Changdao County Marine Ecological Civilization Comprehensive Experimental Zone, Yantai, China

**Keywords:** high-temperature, stress, protein-protein interaction network, *Sepia esculenta*, transcriptome

## Abstract

*Sepia esculenta* is a cephalopod widely distributed in the Western Pacific Ocean, and there has been growing research interest due to its high economic and nutritional value. The limited anti-stress capacity of larvae renders challenges for their adaptation to high ambient temperatures. Exposure to high temperatures produces intense stress responses, thereby affecting survival, metabolism, immunity, and other life activities. Notably, the molecular mechanisms by which larval cuttlefish cope with high temperatures are not well understood. As such, in the present study, transcriptome sequencing of *S. esculenta* larvae was performed and 1,927 differentially expressed genes (DEGs) were identified. DEGs were subjected to functional enrichment analyses using the Gene Ontology (GO) and Kyoto Encyclopedia of Genes and Genomes (KEGG) databases. The top 20 terms of biological processes in GO and 20 high-temperature stress-related pathways in KEGG functional enrichment analysis were identified. A protein-protein interaction network was constructed to investigate the interaction between temperature stress-related genes. A total of 30 key genes with a high degree of participation in KEGG signaling pathways or protein-protein interactions were identified and subsequently validated using quantitative RT-PCR. Through a comprehensive analysis of the protein-protein interaction network and KEGG signaling pathway, the functions of three hub genes (HSP90AA1, PSMD6, and PSMA5), which belong to the heat shock protein family and proteasome, were explored. The present results can facilitate further understanding of the mechanism of high temperature resistance in invertebrates and provide a reference for the *S. esculenta* industry in the context of global warming.

## 1 Introduction

The rapid acceleration of global industrialization has resulted in a significant release of carbon dioxide into the atmosphere, which has led to global warming and subsequent oceanic warming (Nick et al., 2013; Al-Ghussain, 2019; Liu et al., 2020). High temperature will have a strong negative impact on the reproduction and development of marine biological physiological functions (Jones et al., 2013; Li et al., 2016). As an example, prolonged exposure of fish (Vargas-Chacoff et al., 2019), shellfish (Li et al., 2016; Martinez et al., 2018), mollusks (Trigg et al., 2020), and other marine animals to high-temperature marine environments will increase the burden of maintaining physiological activities, and while decreasing their ability to cope with other environmental changes, ultimately affecting their healthy growth. Additionally, high seawater temperatures can lead to stress responses in marine organisms (Dalvi et al., 2017; Samaras et al., 2018). For instance, rising seawater temperatures caused by global warming could impair the physiological function of *Crassostrea virginica* by disrupting the pro-oxidation-antioxidant system (Rahman and Rahman, 2021). When *Piscium* are exposed to high temperatures, the catecholaminergic (norepinephrine and dopaminergic) system is altered, which affects the synthesis, release, and metabolism of neurotransmitters (Alfonso et al., 2021).

*Sepia esculenta* is a commercially significant species belonging to the cuttlefish family, found primarily in the northern seas of China, and possessing high economic value (Guo et al., 2021). In actual aquaculture production, high-temperature seawater will significantly affect the immunity, metabolism, reproduction, and other life activities of *S. esculenta*, which is undoubtedly a significant challenge for *S. esculenta* factory farming (Bian et al., 2018).

In recent years, there has been considerable progress in high-throughput transcriptome sequencing technology, and biological analysis through high-throughput transcriptome sequencing has become more accurate (Morozova et al., 2009; Qian et al., 2014; Bao et al., 2022a; Bao et al., 2022b). To illustrate, through such technology, the expression of heat stress genes in *Pinctada fucata* have been found to be significantly reduced after being stimulated by multiple high temperatures, indicating that *P. fucata* is able to gradually adapt to the impact of high temperatures on their life activities (Li et al., 2016). The larvae possess a relatively weak capacity to endure environmental stress when in their initial phase of growth and development. Thus, elevated seawater temperature significantly impairs the routine life activities of marine larvae (Ginger et al., 2013; Kaplan et al., 2013). In the present study, transcriptome sequencing was performed to explore the mechanism of high-temperature stress in *S. esculenta* larvae.

In the present, high-throughput transcriptome sequencing technology was used to sequence the *S. esculenta* larvae exposed to high temperatures for 0, 4, and 24 h, and the data obtained were mapped to the reference genome of *S. esculenta*. Subsequently, differentially expressed genes (DEGs) were examined using cluster heatmap analysis and subjected to enrichment analyses of GO and KEGG. The protein-protein interaction (PPI) network was constructed by means of the selected DEGs. A combination of the KEGG signaling pathway and the protein-protein interaction network to was innovatively used explore the expression of key genes and families in *S. esculenta* after high-temperature stress. Finally, the expression patterns of key DEGs were verified using quantitative RT-PCR (qRT-PCR). The results provide a reference for further exploration of the temperature stress mechanism of *S. esculenta,* and can be beneficial for the factory farming of *S. esculenta* in terms of facilitating understanding of the negative effects of global warming.

## 2 Materials and methods

### 2.1 Experiment materials

In the present experiment, adult *S. esculenta* samples (weight = 351.87 ± 12.68 g, mantle length = 14.82 ± 0.21 mL) were collected from the marine region near Qingdao, and were held for a period of time in the pool for temporary breeding to ensure the adaptation of adults to the environment. An attachment net was placed in the pool to collect eggs. The temperature at the time of temporary breeding was 21.5°C ± 1°C. The eggs are oval, 7 ± 1 mm in diameter and translucent, which were typically collected every day and placed in perforated plastic pots. Pots were placed in another pool with flowing seawater (dissolved oxygen = 5.5 mg/L, pH = 8.2, and salinity = 30.5 ± 0.3) and continuous oxygenation, and the water temperature and other indicators were the same as those of the parent pool.

### 2.2 Experimental process

In two square 120 L buckets, each bucket was filled with 100 L seawater. The new hatched larvae were transferred to the experimental bucket half an hour after the incubation was completed. The water temperature before the larvae were transferred to the experimental bucket was 21°C ± 1°C. According to prior research, the seawater temperature of the experimental group was set to 28°C, and the temperature of the seawater in the control group was set to 23°C. During the experiment, one hundred larvae of *S. esculenta* were put into each bucket, and samples were taken at 4 and 24 h. Samples at the 0 h time point were obtained individually. All of the samples were stored in cryovials in liquid nitrogen.

### 2.3 RNA extraction and library construction and sequencing

RNA extraction, library construction, and sequencing were supported by Beijing Novogene Company. RNA was extracted from the whole larvae using standard extraction methods, followed by strict quality control with the Agilent 2100 bioanalyzer of RNA samples (Masotti and Preckel, 2006). The kit used for library construction was NEBNext^®^ Ultra™ Directional RNA Library Prep Kit for Illumina^®^. When creating the library, nine larvae were collected from each group at every time point, and three samples were pooled randomly together to form a total of three biological replicates (C_0h_1, C_0h_2, C_0h_3, C_4h_1, C_4h_2, C_4h_3, C_24h_1, C_24h_2, C_24h_3, T_4h_1, T_4h_2, T_4h_3, T_24h_1, T_24h_2, and T_24h_3). *S. esculenta* larvae were sequenced using Illumina NovaSeq 6000 (Illumina, United States).

### 2.4 Gene function annotation and screening of DEGs

In the present study, the structure and function of unigenes were annotated into several databases, including NR, SwissProt, KEGG, GO, Interpro, and PFAM. DEGs were screened out using the DESeq2 package for R as a negative binomial distribution model. First of all, data were imported for building the dds model, and then the DESeq function was used to estimate the dispersion of the samples. Afterwards the difference in gene expressions was analyzed by this package. DEGs with |Log2 Fold Change| ≥ 0 (Love et al., 2014) and *p*-value ≤0.05 were screened out.

### 2.5 Enrichment analyses

DAVID v6.8 (Jiao et al., 2012) was used for GO and KEGG enrichment analyses. DEGs were enriched into GO terms and KEGG signaling pathways to understand the response mechanism of *S. esculenta* after high-temperature exposure.

### 2.6 Protein-protein interaction network

STRING v11.5 (Szklarczyk et al., 2019) was used to construct a PPI network. Genes that play significant roles in response to high-temperature stress in *S. esculenta* larvae were then selected based on the number of PPI and KEGG signaling pathways.

### 2.7 Quantitative RT-PCR validation

Primer Premier 5.0 (Ren et al., 2004) was used to design gene-specific primers (Table 1). In addition, qRT-PCR was performed to validate 30 genes that exhibit significant involvement in response to high-temperature stress in *S. esculenta.* The *β-actin* gene was used as a reference gene for qRT-PCR.

**TABLE 1:** List of primers used for quantitative RT-PCR validation.

Gene name	Forward primer (5′-3′)	TM (°C)	Reverse primer (5′-3′)	TM (°C)	Amplicon length (bp)
*COL12A1*	CGC​AGT​CCT​TGA​GAA​CAT​AG	60	CGT​TGT​AGT​CGT​CGT​TGT​AG	60	157
*COL15A1*	GTC​ATC​CAG​TTG​GGT​GTT​AG	60	CAG​TGT​CCG​AGC​AGA​TTA​TAC	60	149
*COL6A3*	ACGGCAGAACGAACAATC	60	CAC​CTT​TCA​TGT​CCA​CTA​CTC	60	108
*COL6A5*	AGAGCGCAACCATTCATC	60	GAA​TAG​ACG​TCT​CGA​CAA​GC	60	102
*COL6A6*	GCA​GTT​CTG​ATC​TCG​TCT​TT	60	GGT​GAC​TTC​CTT​GAT​GTC​TG	60	101
*DNAJA1*	GCT​GGT​GAA​GGA​GAT​CAA​TG	60	GAT​CAT​CAC​CCT​GTC​GTT​TG	61	100
*DNAJC10*	CAT​CAG​CTC​CTT​GGG​TAA​TC	60	GCT​TCC​AAC​ATT​CAC​CAA​AC	60	110
*HSP90AA1*	CAA​CAC​CCT​GAC​CCT​TAT​TG	60	CCA​CAA​GGT​AAG​CCG​AAT​AG	60	176
*HSPA8*	CCT​TCT​CCT​CTT​GGA​TGT​TG	60	GCT​TGG​TAG​GAA​TGG​TTG​T	60	101
*HYOU1*	GGA​AGT​CTT​TCA​GCT​CCT​TG	60	GCA​CGG​ACA​CCT​TTG​TAT​T	60	103
*ODC1*	ACATGGGAGCCTACACTT	60	CACCACACAACGGGATTT	60	116
*PIK3R1*	CAG​TTT​GTA​GTC​GGG​AAA​GAG	60	TAAAGCAGCCAGCCAATC	60	104
*PIK3R4*	CTT​CCA​ACT​GCC​TAC​CTT​TC	60	CGA​GTG​CTT​ATT​CGG​TCA​TAC	60	103
*PSMA2*	GCT​ACT​GCA​ATG​GGT​AAG​AA	60	ACT​CTC​CTT​CAG​GGT​TAA​GA	60	117
*PSMA4*	CCT​CGG​ATG​GTG​TTC​TCT​TA	61	CAA​CAC​TGC​AAG​CCA​TAT​CA	61	114
*PSMA5*	CTC​ACC​AGA​AGG​CAG​ATT​G	60	CAA​CAC​GAC​ACC​CTC​ATT​T	60	100
*PSMA6*	CAC​TTG​GGT​GCT​GTA​TGA​TT	60	CAGCAGCAGTGGCTTTAT	60	108
*PSMA8*	GCA​AAG​ACT​GTT​CGT​GAG​TA	60	CCA​GAT​TGG​ACC​ACT​TCT​AAC	60	113
*PSMB2*	CAT​GGC​TAT​GGT​GCC​TAT​TT	60	CAT​GGA​AGG​AGG​GAA​GAT​TG	60	145
*PSMB5*	CCC​ACG​CTT​ATG​GTG​TAT​TG	61	CAT​CTC​TGT​GTG​TTG​CAT​GA	60	105
*PSMB6*	TCA​GAG​TAG​CAG​CCC​ATA​TC	61	CCT​GTC​CTC​CTC​TGT​GTT​TA	61	105
*PSMC3*	CTC​AGG​ACC​AAG​AAG​AAG​AAG	60	CCA​ATG​ACA​GGC​AGG​AAA​TA	60	115
*PSMD1*	CTG​CCC​AGC​AAG​ACA​TAT​T	60	CCA​GCA​TTA​CAA​GAC​CCA​TAG	60	102
*PSMD12*	CCA​GGA​GGC​TTG​TTC​TTA​TG	60	GTG​TAA​GCC​GAG​CTC​TTT​C	60	125
*PSMD2*	CCCAGCCAAGGCTTTATT	60	TCT​GTT​GGA​ACC​AGC​ATA​AG	60	103
*PSMD3*	ATT​GTC​GCC​AAG​GCT​ATT​C	60	GGC​TCT​CTG​GTT​GAG​TAA​ATG	60	107
*PSMD6*	AGT​CTT​ACC​GAA​GCC​TTA​CT	60	GCGTCCAGCAGCAATAAA	61	101
*PSMD7*	GGT​TTG​TGG​AGT​ACT​ACT​TG	60	GTC​CAG​AAA​CCA​CAC​AGA​TT	60	112
*PSMD8*	AAGCAGAGCCATCGAAAC	60	CGG​GCA​GAT​TAT​CCT​TGT​AG	60	153
*SUGT1*	GAG​AAG​GCA​ATC​GTG​GAT​AC	60	GGG​CGT​CTT​GGT​AAT​TCT​T	60	112

## 3 Results

### 3.1 Sequencing results and quality

High-throughput sequencing technology was used to sequence the samples of *S. esculenta* larvae. On average, 87.68% of the high-quality reads were able to be aligned to the reference genome, and the percentage of high-quality reads with a Q30 score was 93.04% (Table 2). Such data indicate that the sequencing quality of all samples was adequate for subsequent analysis.

**TABLE 2:** RNA-Seq data.

Sample	Raw reads	Clean reads	Clean reads Q30 (%)	Clean reads Q20 (%)	Total mapping	Mapping rate (%)
C_0h_1	44,822,088	44,401,358	93.02	97.42	38,945,045	87.71
C_0h_2	46,604,268	46,067,346	92.97	97.39	40,192,257	87.25
C_0h_3	42,199,716	41,745,596	92.31	97.08	35,955,570	86.13
C_4h_1	42,594,570	42,050,900	93.35	97.56	37,123,254	88.28
C_4h_2	45,122,216	44,583,624	92.89	97.37	39,143,445	87.80
C_4h_3	43,910,186	43,339,204	93.00	97.44	37,996,122	87.67
C_24h_1	44,237,100	43,609,904	93.06	97.45	38,548,116	88.39
C_24h_2	45,963,126	45,180,404	93.07	97.49	39,479,438	87.38
C_24h_3	45,268,732	44,022,418	93.27	97.58	38,685,844	87.88
T_4h_1	44,626,628	44,162,538	93.06	97.48	38,927,372	88.15
T_4h_2	42,318,638	41,872,474	93.24	97.53	36,828,643	87.95
T_4h_3	40,793,398	40,357,954	92.99	97.40	35,682,178	88.41
T_24h_1	41,849,070	41,155,332	93.09	97.43	36,127,995	87.78
T_24h_2	45,764,258	44,923,156	93.11	97.48	39,397,933	87.70
T_24h_3	46,116,390	45,123,238	93.10	97.47	39,109,229	86.67

### 3.2 Gene function annotation

A total of 32,138 genes were annotated to different databases, and most genes were annotated to the NR database, reaching 73.67% (Table 3). At the same time, a large number of genes were annotated in the SwissProt and KEGG databases.

**TABLE 3:** Gene function annotations.

	Number of genes	Percentage (%)
Annotated in NR	23,677	73.67
Annotated in SwissProt	19,757	61.48
Annotated in KEGG	16,509	51.37
Annotated in GO	1,788	5.56
Annotated in Interpro	9,032	28.10
Annotated in PFAM	6,879	21.40
Total unigenes	32,138	100.00

### 3.3 Differential gene expression analysis

#### 3.3.1 Volcano plot of DEGs

**FIGURE 1:**
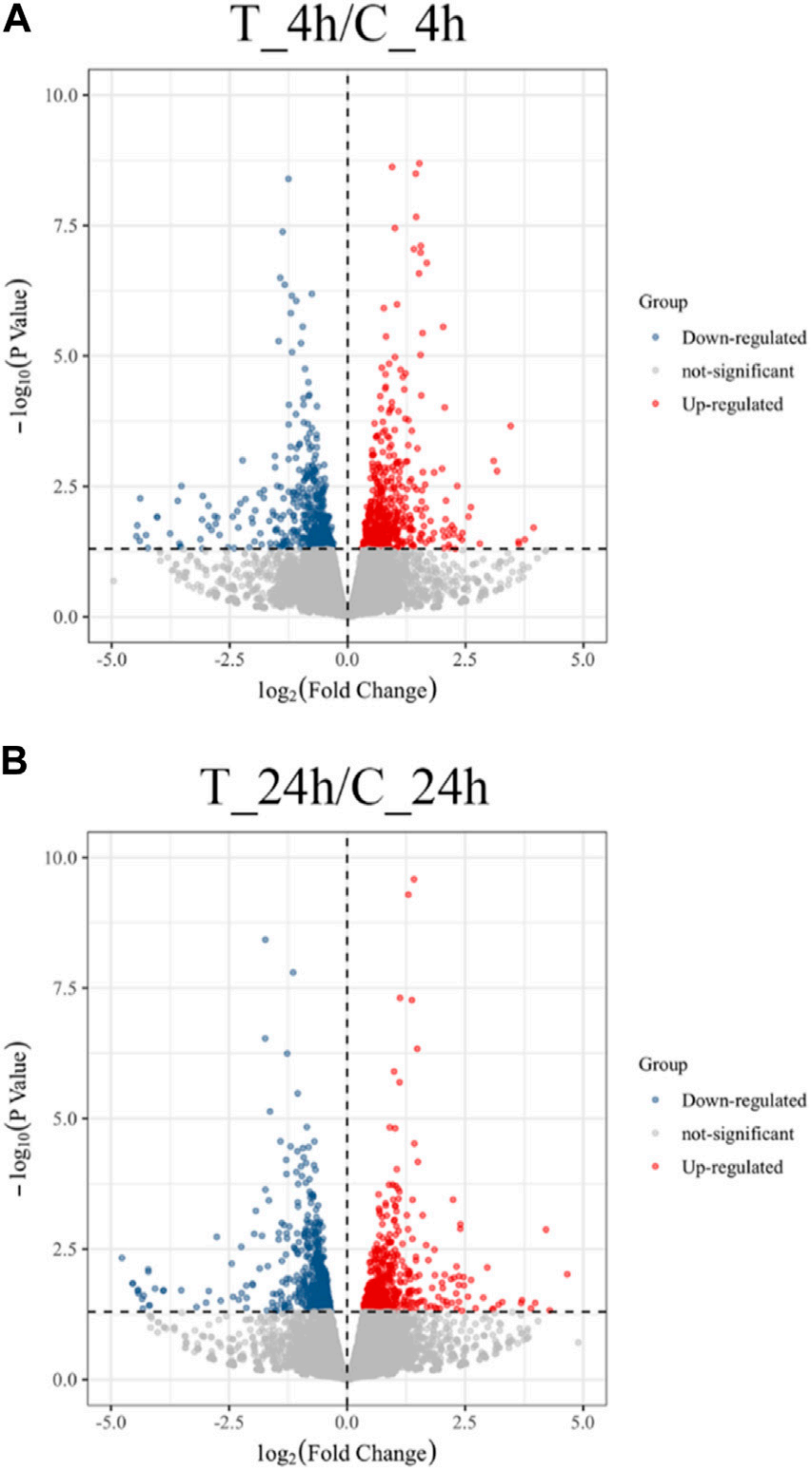
Volcano plot of the distribution expression of DEGs. **(A)** DEGs expression distributions between T_4h/C_4h. Red dots represent upregulated DEGs; blue dots are downregulated DEGs; grey dots indicate non-significantly genes. **(B)** DEGs expression distributions between T_24h/C_24h. Each dot represents a gene.

The volcano plot shows that 991 DEGs were identified, of which 498 DEGs were upregulated and 493 were downregulated in the 4 h exposure sample. In the 24 h exposure sample, 1,064 DEGs were identified among which, 470 DEGs were upregulated, and 594 DEGs were downregulated (Figure 1).

#### 3.3.2 Venn diagram analysis of DEGs

**FIGURE 2:**
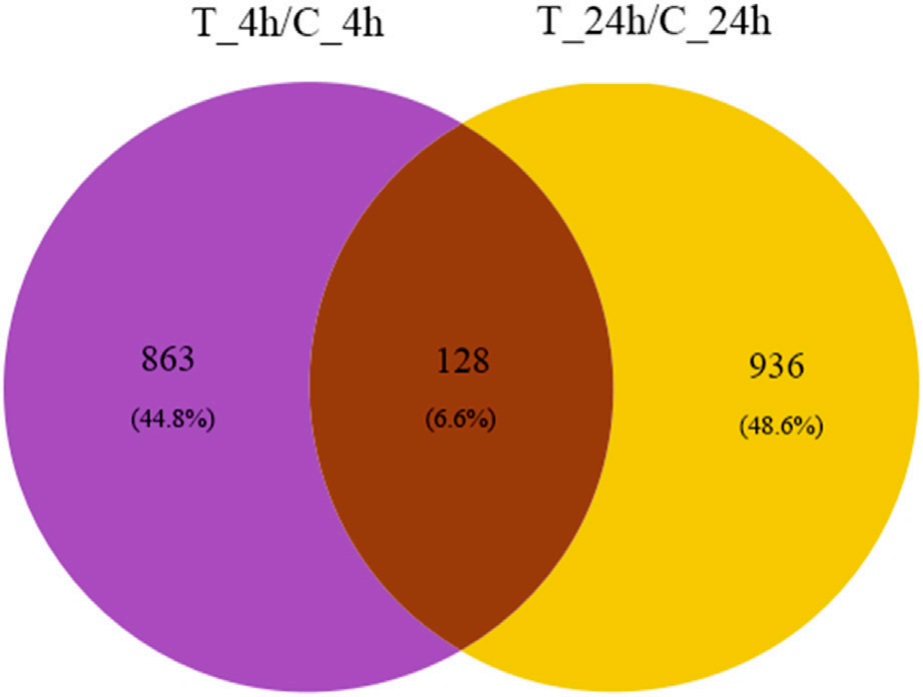
Venn diagram of DEGs. The purple and yellow regions represent the number of genes significantly expressed at 4 and 24 h after exposure, respectively. The number of genes co-expressed at 4 and 24 h is represented by brown areas.

A total of 1,927 genes were differentially expressed, of which 128 genes were co-expressed at both 4 and 24 h (Figure 2).

#### 3.3.3 Cluster heatmap analysis

**FIGURE 3:**
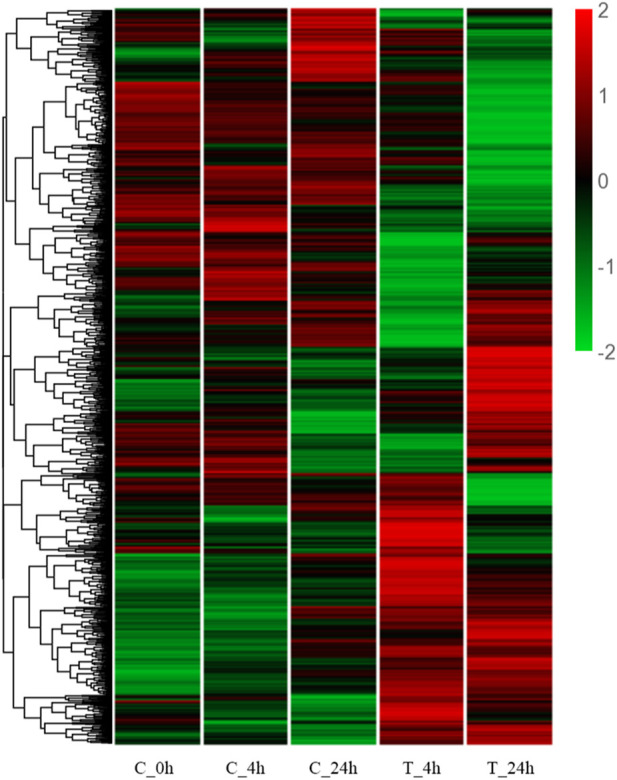
Hierarchical clustering heatmap of DEGs. The Y-axis represents each gene, and the X-axis represents the different treatment groups. Colors ranging from green to red indicating low to high expression levels.

The cluster heatmap (Figure 3) shows that the blank control group (C_0h), the 4 h control group (C_4h), and the 24 h control group (C_24h) had basically the same expression pattern. Compared with the 4 h control group (C_4h), the gene expression patterns in the 4 h experimental group (T_4h) were significantly different. A different trend was observed in the two groups (C_24h) (T_24h) at 24 h.

### 3.4 Functional enrichment analyses of GO and KEGG

**FIGURE 4:**
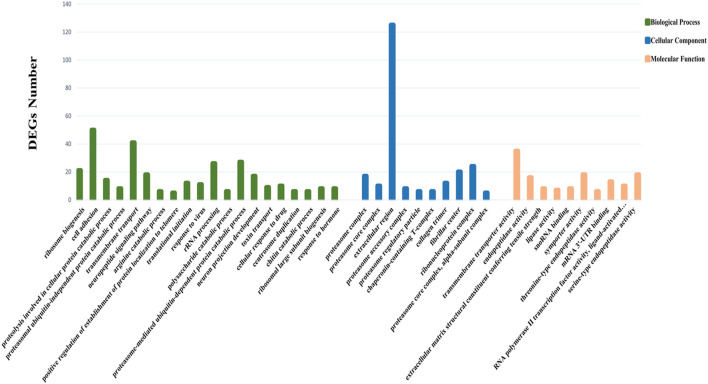
GO enrichment analysis of DEGs. The Y-axis represents the number of DEGs enriched to this term; the X-axis stands for the specific terms based on GO.

**FIGURE 5:**
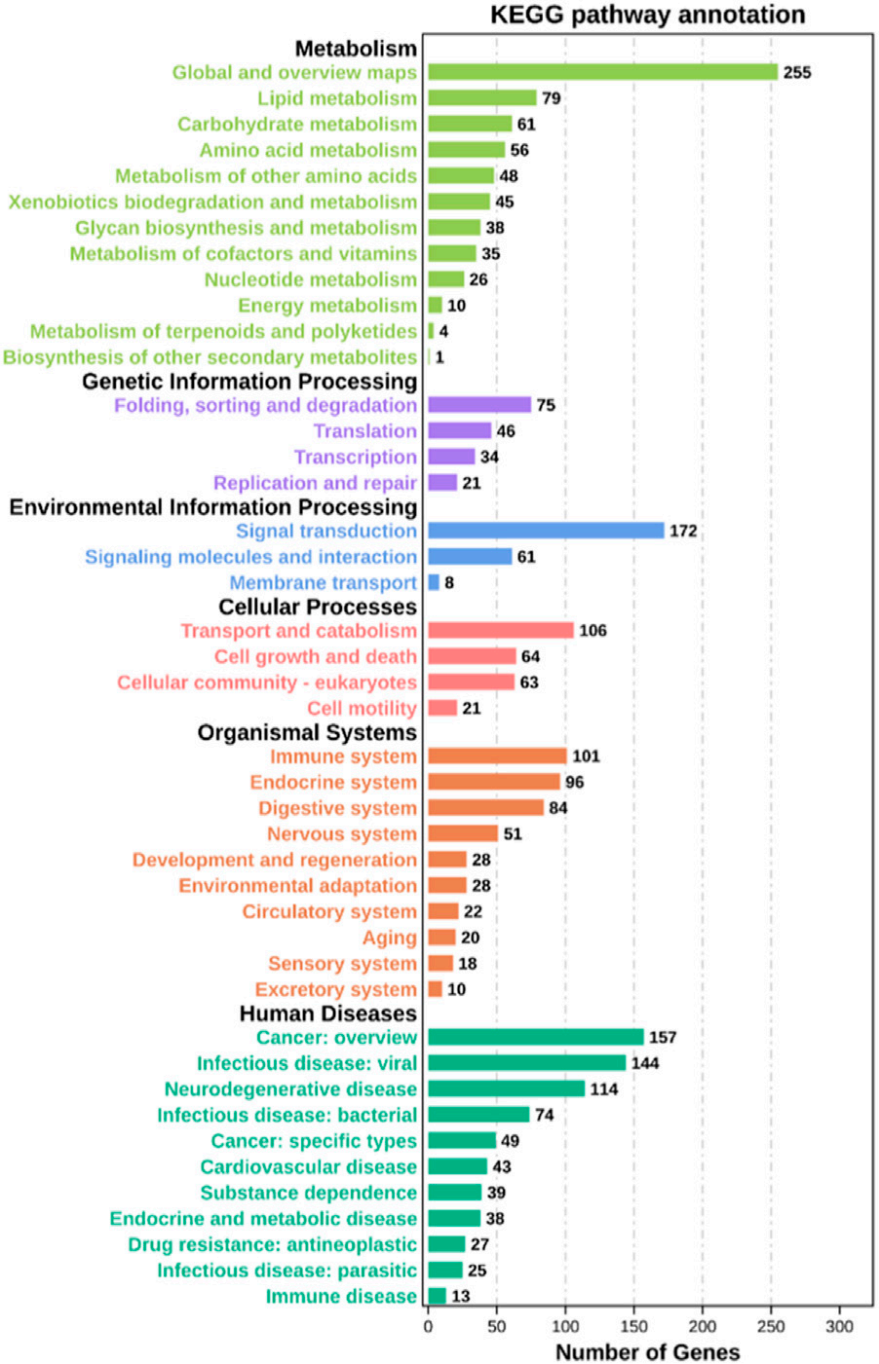
Level-2 KEGG signaling pathway annotation. The Y-axis indicates level-2 KEGG classes; the X-axis represents the corresponding number of DEGs.

Through GO functional enrichment analysis, DEGs were enriched into three categories: biological process, cellular component, and molecular function. The top 20 terms of the biological process and the top 10 terms of the cellular component and molecular function were selected (*p*-value ≤ 0.05) (Figure 4). A large number of the top 20 terms in the biological process were associated with high-temperature stress. The KEGG enrichment analysis results indicate that a large number of DEGs were enriched in multiple level-2 KEGG signaling pathways (Figure 5). Further, 20 level-3 KEGG signaling pathways were significantly enriched after exposure to high temperatures (Table 4).

**TABLE 4:** Twenty significantly enriched KEGG signaling pathways related to high temperature exposure.

Pathway	Number of DEGs
Antigen processing and presentation	4
Apelin signaling pathway	9
Arginine and proline metabolism	6
Arginine biosynthesis	3
ECM-receptor interaction	7
FoxO signaling pathway	5
MAPK signaling pathway	6
Neurotrophin signaling pathway	3
NOD-like receptor signaling pathway	6
PI3K-Akt signaling pathway	8
Proteasome	16
Protein digestion and absorption	9
Protein processing in endoplasmic reticulum	9
Ras signaling pathway	3
Relaxin signaling pathway	3
Retinol metabolism	3
Ribosome biogenesis in eukaryotes	5
RNA degradation	6
Steroid hormone biosynthesis	4
Transcriptional misregulation in cancer	11

Among the 20 significantly enriched KEGG signaling pathways after high-temperature exposure, a total of 97 DEGs were enriched. Such genes that are involved in multiple high-temperature stress-related signaling pathways may be a significant factor in the process of resisting high-temperature stress in *S. esculenta*.

### 3.5 Construction of protein-protein interaction network

**FIGURE 6:**
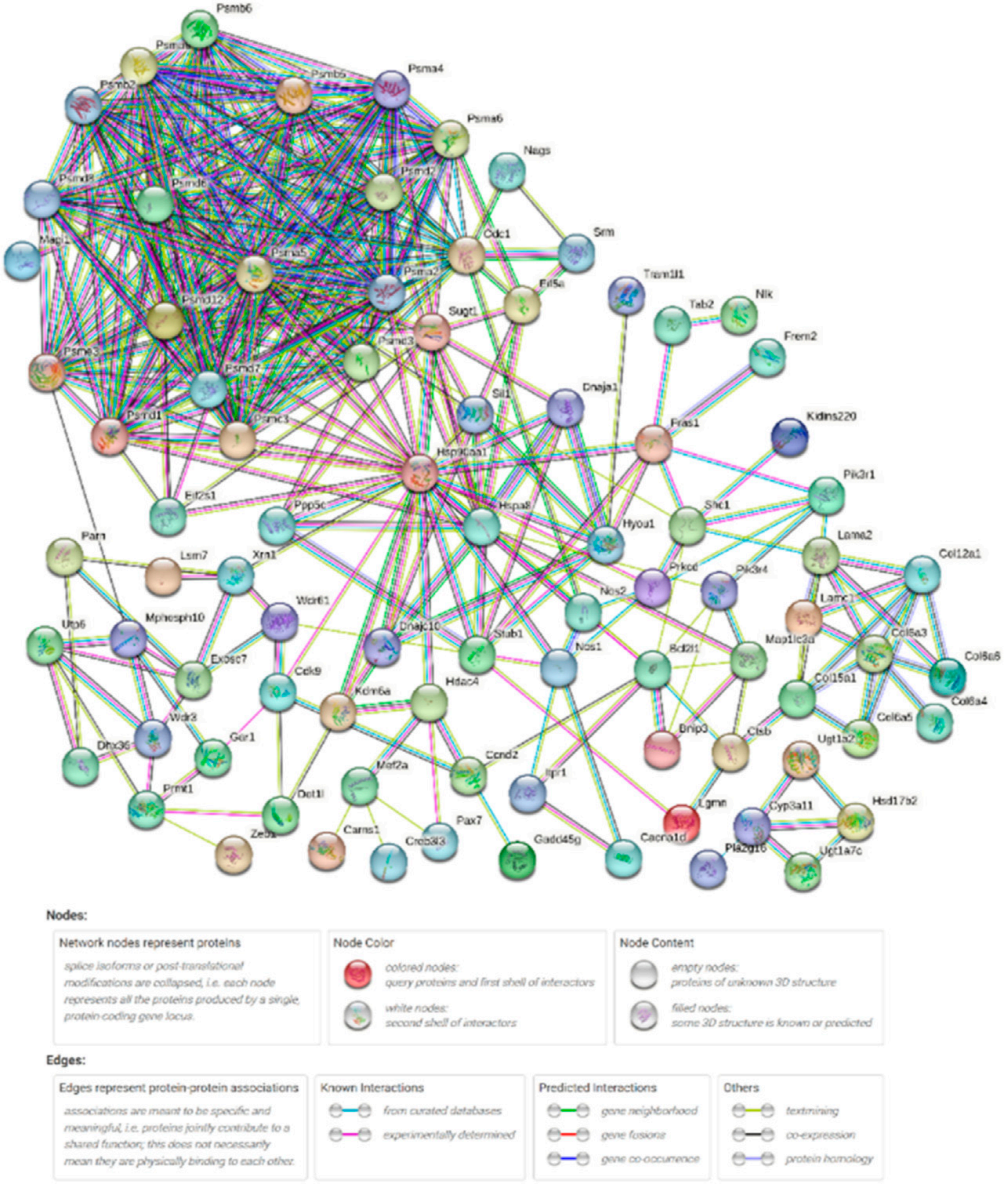
High-temperature stress-related PPI network. Network nodes represent proteins. Legends represent relationships between nodes.

In the present study, 97 DEGs (Supplementary Table S1) enriched in the KEGG pathways identified after high-temperature exposure were used to construct a PPI network (Figure 6), thereby facilitating identification of key genes after high-temperature exposure.

Table 5 shows the network parameters. The average node degree was 6.96, the clustering coefficient was 0.574 and the PPI enrichment *p*-value was 1.0E-16. The parameters indicate significant interactions between the above DEGs.

**TABLE 5:** Statistics of temperature stress response-related PPI network parameters.

Network stats	
Number of nodes	92
Number of edges	320
Average node degree	6.96
Clustering coefficient	0.574
Expected number of edges	115
PPI enrichment *p*-value	1.0E-16

### 3.6 Selection of key high temperature stress response genes

After exposing *S. esculenta* larvae to high temperature, there was a significant level of interaction observed among the DEGs. The number of protein interactions was used as the main reference factor, combined with the number of KEGG signaling pathway participation to select the key genes. Finally, 30 key genes were identified. Table 6 displays the 30 identified key DEGs, along with their corresponding numbers of PPI and KEGG signaling pathways.

**TABLE 6:** Thirty key DEGs and their corresponding number of PPI and KEGG signaling pathways.

Gene name	Number of protein-protein interaction	Number of KEGG signaling pathway
*COL12A1*	6	1
*COL15A1*	6	1
*COL6A3*	7	3
*COL6A5*	3	3
*COL6A6*	3	3
*DNAJA1*	5	1
*DNAJC10*	5	1
*HSP90AA1*	26	1
*HSPA8*	14	2
*HYOU1*	7	1
*ODC1*	21	1
*PIK3R1*	5	1
*PIK3R4*	5	1
*PSMA2*	19	1
*PSMA4*	18	1
*PSMA5*	21	1
*PSMA6*	19	1
*PSMA8*	17	1
*PSMB2*	17	1
*PSMB5*	17	1
*PSMB6*	17	1
*PSMC3*	18	1
*PSMD1*	20	1
*PSMD12*	20	1
*PSMD2*	18	1
*PSMD3*	19	1
*PSMD6*	21	1
*PSMD7*	18	1
*PSMD8*	17	1
*SUGT1*	11	1

The 30 key DEGs could be further divided into five categories based on their families and functions, including the PI3K-Akt signaling pathway, heat shock protein family, proteasome family, collagen family, and other genes regulating high temperature stress.

### 3.7 Validation of key DEGs using quantitative RT-PCR

**FIGURE 7:**
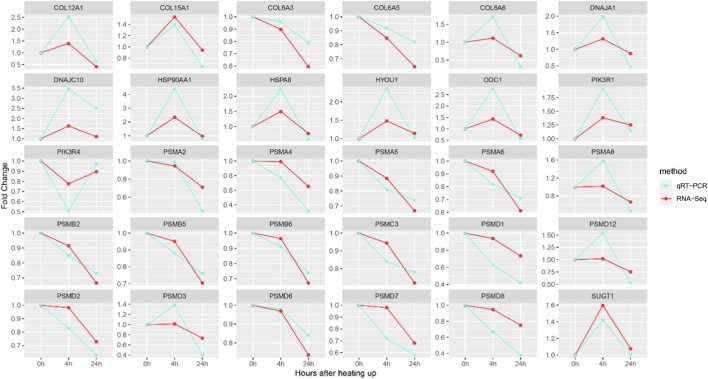
qRT-PCR and RNA-Seq results of key DEGs. The Y-axis represents the fold change and the X-axis represents the time of high temperature exposure.

The accuracy of the expression of 30 key genes was verified using qRT-PCR. The expression patterns of genes obtained from qRT-PCR were consistent with those obtained from RNA-Seq analysis, suggesting that the RNA-Seq results are reliable (Figure 7).

## 4 Discussion

### 4.1 Expression and functional enrichment analysis of DEGs

In the present study, 1,927 DEGs that play significant roles in the resistance to high-temperature stress in the *S. esculenta* were screened. Volcano plot analysis showed that more genes appeared differentially expressed as the duration of high-temperature exposure increased. Cluster heatmap analysis showed that a large number of DEGs showed different expression patterns at 4 h (T_4h) and 24 h (T_24h). A total of 1,927 DEGs were used for GO and KEGG functional enrichment analysis. The majority of the top 20 terms identified in the cluster of biological processes, as revealed by the GO enrichment analysis, were associated with the response to high-temperature stress. The significantly enriched transmembrane transport identified through GO enrichment analysis could enhance material exchange between organelles and effectively alleviate cell stimulation (Schendzielorz et al., 2018; Jones et al., 2019; Zhong and Zhao, 2019; Ming et al., 2020). Proteasome ubiquitination, a process that helps to remove damaged proteins to reduce cell damage caused by external stimulus, was downregulated at 24 h (Shringarpure et al., 2001; Myers et al., 2018). The described biological processes may be significant factors in larval stress following high temperature exposure. In addition, among the 20 significantly enriched KEGG signaling pathways, the PI3K-Akt signaling pathway and MAPK signaling pathway play a crucial role in mollusk immunity (Song et al., 2005; Kharchenko et al., 2010; Vergadi et al., 2017; Wang et al., 2019; Qiao et al., 2021; Bao et al., 2022a). The immune response of *S. esculenta* larvae might be activated to protect against the injury caused by high temperature exposure. The results of GO and KEGG functional enrichment analyses show that the larvae of *S. esculenta* might have an intense stress response after being stimulated by high-temperature seawater. The analysis of GO terms and KEGG pathways can facilitate comprehensive understanding of the response mechanisms of *S. esculenta* larvae to temperature stress, which is beneficial for actual artificial breeding.

### 4.2 Analysis of PPI network

The growth and development of organisms, and metabolism, immunity, and other physiological functions are inseparable from the participation of proteins, which are the basis of all life activities (Ahmad et al., 2021; Chandhini et al., 2021; Li et al., 2021). The analysis of the interaction between proteins facilitates further understanding of the resisting mechanism of high-temperature stress in *S. esculenta* larvae. In the present study, a PPI network was constructed using 97 high-temperature stress-related DEGs in 20 significantly enriched KEGG signaling pathways. The results of the network show that there were strong interactions between proteins. For instance, HSP90AA1 (Heat shock protein 90 alpha family class A member 1), PSMD6 (Proteasome 26S subunit, non-ATPase 6), PSMA5 (Proteasome 20S subunit alpha 5), and ODC1 (Ornithine decarboxylase 1) interacted with over 20 proteins. Therefore, the suggestion of the present authors is that the aforementioned proteins may play central roles in the mechanism of high-temperature stress in *S. esculenta* larvae. Further investigation is necessary to elucidate the mechanism of action of the identified key genes involved in the response to high-temperature stress in *S. esculenta* larvae.

### 4.3 Analysis of critical pathways and families

In the present study, transcriptome sequencing technology was used to analyze the stress reaction of *S. esculenta* larvae in high-temperature seawater, and the results can facilitate further understanding of to further understand the stress mechanism of high-temperature resistance in larvae. Finally, 30 key genes with a high number of interactions or a high number of KEGG signaling pathway involvement were identified.

#### 4.3.1 PI3K-Akt signaling pathway regulates immune defense

The PI3K-Akt signaling pathway plays a central role in immune regulation by controlling the proliferation, differentiation, and migration of immune cells (Song et al., 2005; Vergadi et al., 2017; Bao et al., 2022a). Previous studies have shown that the PI3K-Akt signaling pathway in mollusks is involved in and regulates immune processes (Canesi et al., 2002; Fukao and Koyasu, 2003; Troutman et al., 2012). For example, the PI3K-Akt signaling pathway can regulate phagocytosis during the immune response elicited by organismal stimulation. Further, the pathway can induce an immune defense response and control the apoptosis and growth of immune cells following environmental stimulation (Sun et al., 2016; Yu et al., 2019; Wang et al., 2020). In the present study, two genes including PIK3R1 (Phosphoinositide-3-kinase regulatory subunit 1) and PIK3R4 (Phosphoinositide-3-kinase regulatory subunit 4) were identified. As previously reported, environmental stimuli can down-regulate the expression of PIK3R1, which in turn activates the mitochondrial apoptosis and death receptor pathways (Yin et al., 2020). PIK3R4 expression promotes the formation of autolysosomes for protein degradation (Gámez-Díaz et al., 2022). The results suggest that the PI3K-Akt signaling pathway may play a significant role in the resistance of *S. esculenta* larvae to high-temperature stress. We speculated that the PI3K-Akt signaling pathway may induce the activation of immune signaling factors to induce immune defense in larvae when stimulated by high temperature. At the same time, the expressions levels of PIK3R1 and PIK3R4 enriched in this pathway were significantly downregulated, which may also induce the apoptotic pathway to alleviate the damage of high-temperature exposure.

#### 4.3.2 Heat shock protein repair damage

**FIGURE 8:**
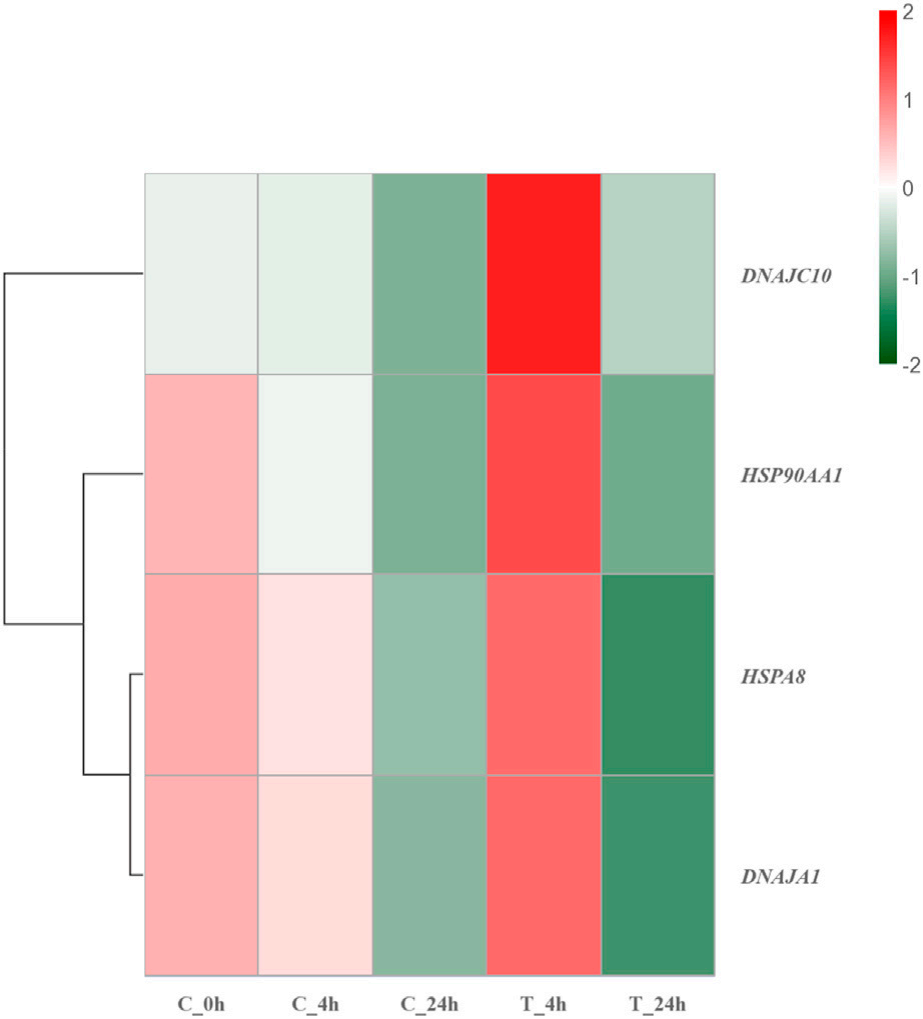
Hierarchical clustering heatmap of HSP family genes. Colors ranging from green to red indicating low to high expression levels.

Heat shock proteins are a class of chaperone proteins that is widely present in various organisms and is expressed in large quantities in response to high temperature stimulation (Brokordt et al., 2009; Zuehlke et al., 2015; Wu et al., 2017). The main function of heat shock proteins is to assist in the refolding of misfolded proteins, as well as to remove damaged amino acid chains that cannot be properly folded and to degrade damaged proteins. Such actions contribute to maintaining protein homeostasis within organisms (Sghaier et al., 2004; Brun et al., 2008; Kumar et al., 2022). At the same time, HSPs can also improve the resistance to stress in the organism. Previous studies have indicated that the energy metabolism and antioxidant capacity of *Ruditapes philippinarum* can be improved after a brief high-temperature treatment (Zhang et al., 2023). In the present study, HSP90AA1, HSPA8 [Heat shock protein family A (HSP70) member 8], DNAJC10 [DNAJ heat shock protein family (HSP40) member C10], and DNAJA1 [DNAJ heat shock protein family (HSP40) member A1] in the HSPs family had strong interactions with other proteins, and were significantly upregulated in 4 h after high-temperature stress. HSP90AA1 had the highest number of PPI. Previous studies have linked HSP90AA1 to protein trafficking, transcriptional regulation of gene expression, and epigenetic processes (Csermely et al., 1998; Wegele et al., 2004). HSP90AA1 is sensitive to temperature and can respond quickly to high-temperature stimulation, being a soluble protein of the HSPs family, which mainly exists in the cytoplasm (Pearl and Prodromou, 2006; Swirplies et al., 2019). Similar to the functions of other HSPs family genes, HSP90AA1 can bind to hydrophobic fragments of the proteasome, thereby facilitating generation and removal of damaged proteins (Kuckelkorn et al., 2000; Gouy and Delmotte, 2008; Hayashi and Kamikawa, 2011; Reeg et al., 2016). However, differing from other chaperone proteins, HSP90AA1 has a high binding specificity (Lawless et al., 2013). HSPA8, with the participation of ATP, binds to the hydrophobic fragment of the newly generated protein to help the protein fold correctly (Stricher et al., 2013). Deletion of the gene causes selective tissue deformities during embryonic development (Wang et al., 2022). DNAJC10 and DNAJA1 belong to small molecule HSPs (HSP40s), which can bind to HSP70s through the J domain to enhance the interactions between HSP70s and substrates (Qiu et al., 2006; Liu et al., 2020). HSP40s are involved in immune defense when the organism is stimulated (Yan et al., 2021). Figure 8 shows that the genes of HSPs were significantly upregulated after 4 h of exposure to high temperature, while the genes of the HSPs were significantly downregulated after 24 h of exposure. Such findings could be attributed to prolonged exposure to high temperatures causing damage to the larval stress resistance system, leading to inadequate expression of HSPs. Based on the described results, macromolecular HSPs, HSP90AA1 and HSPA8, may protect against protein damage caused by high temperatures by binding to newly generated proteins to help them fold correctly. Small molecule HSPs, DNAJC10 and DNAJA1, may combine with HSP70s to help the protein fold correctly and improve the heat resistance of the organism. At the same time, the anti-stress mechanism of *S. esculenta* larvae was activated by HSPs families to alleviate the damage caused by high temperature stimulation. Extended exposure to high temperature can surpass the threshold of the anti-stress system, resulting in a decrease or impairment of the system.

#### 4.3.3 Proteasome function is disrupted

**FIGURE 9:**
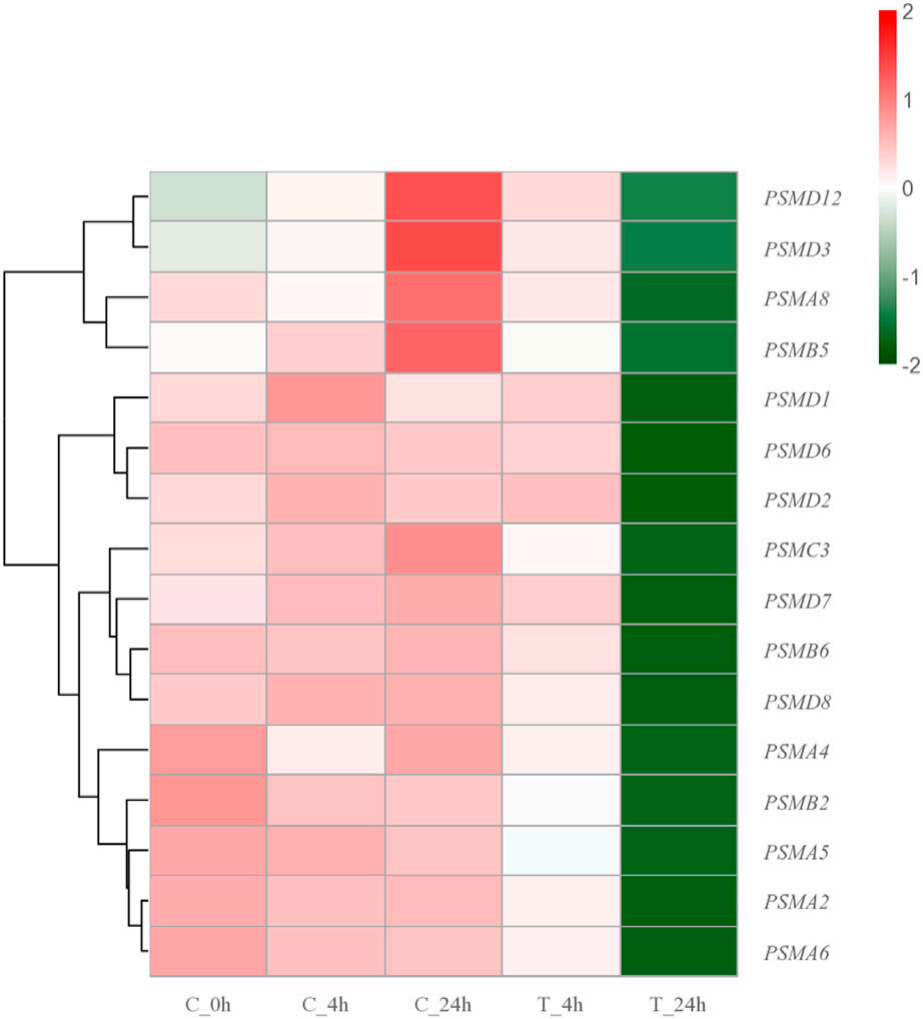
Hierarchical clustering heatmap of proteasome family genes. The colors ranging from green to red indicate low to high expression levels.

The proteasome is a large cylindrical protein complex that primarily degrades unwanted or damaged proteins by cleaving peptide bonds (Morozov et al., 2019; Račková and Csekes, 2020). Such function is the primary mechanism through which cells regulate the concentrations of specific proteins and remove misfolded proteins (Shin et al., 2020; Wang et al., 2020). The components of the proteasome family include 20s core particles, 19s regulatory particles, and 11s regulatory particles (Raynes et al., 2016). Previous studies have reported significant upregulation of genes encoding proteasome and antioxidant enzymes in *Chlamys opercularis* when exposed to the toxin domoic acid (Ventoso et al., 2019). Similarly, genes involved in the ubiquitin-proteasome pathway were found to be significantly upregulated after *R. philippinarum* infection with *Vibrio anguillarum* (Lin et al., 2022). Among the identified key DEGs in the present study, PSMCs and PSMDs belong to the 26s proteasome, which consists of a 20s core granule and two 19s regulatory granules. PSMD6, a member of the 26S proteasome, had the highest number of PPI within the top three. During the degradation of proteins damaged by high-temperature stimulation by the 26S proteasome, the small molecule protein ubiquitin is required to covalently link the degraded proteins in a process called ubiquitination (Saeki, 2017; Shin et al., 2020). The ubiquitinated protein is then recognized by the 19S regulatory particles, which requires the participation of ATP, and the degradation of the protein is conducted by the β subunit in the 20S core particle (Saeki, 2017; Shin et al., 2020; Bhat et al., 2022). When the high-temperature stress response occurs, heat shock proteins are abundantly expressed. HSPs can bind to the hydrophobic regions of misfolded proteins to guide the conjugation of ubiquitin to misfolded proteins and improve the degradation efficiency of proteasomes (Kuckelkorn et al., 2000; Reeg et al., 2016). The PSMAs and PSMBs identified in the genes belong to the 20S proteasome, which contains only one 20S core particle. PSMA5 belongs to the category of 20S proteasome and is one of the top three genes in the number of PPI. The 20S complex can also act alone to degrade damaged proteins when the organism is acutely stimulated (Shringarpure et al., 2001; Myers et al., 2018; Abi Habib et al., 2020; Sahu et al., 2021). The proteasome plays a central role in removing erroneous proteins and maintaining cell stability in *S. esculenta* larvae when stimulated by high temperatures. In contrast to the expression trend of HSPs family genes, genes of the proteasome family were consistently downregulated with increasing exposure time after high-temperature exposure (Figure 9). In consideration of the fact that the main function of proteasomes is to degrade erroneous proteins, we hypothesized that proteasomes should operate at normal temperature rather than excessive temperature (28°C). Exposure to high temperatures can lead to a malfunction of the proteasome-ubiquitin system. Meanwhile, prolonged exposure to high-temperature can cause more serious damage to the anti-stress system of *S. esculenta* larvae, which in turn leads to the reduction of gene expression.

#### 4.3.4 Collagen involved in tissue repair

Collagen is a structural protein containing a triple helical domain that plays a central role in maintaining cellular organization (Ricard-Blum and Ruggiero, 2005; Ricard-Blum, 2011). Collagen also has other vital roles, such as being involved in cell adhesion, chemotaxis and migration, as well as regulating wound healing and tissue remodeling (Brown and Timpl, 1995; Myllyharju and Kivirikko, 2004). Previous research has suggested that HSPs play a role in regulating collagen synthesis induced by transforming growth factor-β, possibly through modulation of Smad 2/3 phosphorylation (Lee et al., 2016). Additionally, HSPs have the ability to bind to newly synthesized collagen and assist in proper folding, resulting in the formation of a stable triple helix structure (Nagata, 1996; Mala and Rose, 2010). In the present study, the associated genes enriched to encode the proteasome included COL6A3 (Collagen type VI alpha 3 chain), COL12A1 (Collagen type XII alpha 1 chain), COL15A1 (Collagen type XV alpha 1 chain), COL6A5 (Collagen type VI alpha 5 chain), and COL6A6 (Collagen type VI alpha 6 chain). The expression levels of such genes were significantly upregulated after 4 and 24 h of exposure. The results suggest that collagen is a significant factor in the resistance of *S. esculenta* larvae to high temperature stress. It is hypothesized that collagen is involved in tissue repair and facilitate repair and regeneration of cells after high temperature stress.

### 4.4 Other high-temperature stress related DEGs

ODC1, SUGT1 (SGT1 homolog, MIS12 kinetochore complex assembly cochaperone), and HYOU1 (Hypoxia upregulated 1) were also identified as genes that have significant roles in the stress resistance mechanism of *S. esculenta* larvae after high-temperature exposure. The significant downregulation of ODC1 can mitigate the apoptosis and damage resulting from exposure to high temperatures in the organism (Jeffries et al., 2012; Jiang et al., 2018). Additionally, SUGT1 can activate the NOD-like receptor family to eliminate damaged proteins, thereby counteracting the negative impacts of high-temperature stress (da Silva Correia et al., 2007; Hong and Hahn, 2016). The expression product of the HYOU1 gene can activate the PI3K-AKT signaling pathway to promote cell growth and migration (Li et al., 2019; Wang et al., 2022). Such genes were all significantly downregulated, indicating that excessive temperature would destroy the anti-stress system of *S. esculenta* larvae and reduce the expression of proteins.

## 5 Conclusion

In the present study, transcriptome sequencing technology was used to preliminarily analyze the mechanism of high-temperature stress in *S. esculenta* larvae. Through the analysis of functional enrichment and protein-protein interaction networks, a significant number of DEGs that play crucial roles in response to stress were identified. The expression trends of key genes in the heat shock protein family and proteasome family indicated that prolonged high-temperature exposure would disrupt the larval stress system. In conclusion, high-temperature exposure could significantly affect the *S. esculenta* larvae stress system, and even prolonged high temperature could cause severe damage to the stress system. The obtained results offer valuable insights to investigate the mechanism underlying the stress response of cephalopods upon exposure to high temperatures.

## Data Availability

The datasets presented in this study can be found in online repositories. The names of the repository/repositories and accession number(s) can be found below: https://www.ncbi.nlm.nih.gov/bioproject/PRJNA947123.
